# Optimising motor learning in infants at high risk of cerebral palsy: a pilot study

**DOI:** 10.1186/s12887-015-0347-2

**Published:** 2015-04-01

**Authors:** Catherine Morgan, Iona Novak, Russell C Dale, Nadia Badawi

**Affiliations:** School of Medicine, University Of Notre Dame Australia, Darlinghurst, NSW Australia; Cerebral Palsy Alliance Research Institute, University of Notre Dame Australia, Darlinghurst, NSW Australia; Department of Neurology, Children’s Hospital at Westmead, University of Sydney, Sydney, Australia; Grace Centre for Newborn Care, Children’s Hospital at Westmead, University of Sydney, Sydney, Australia

**Keywords:** Cerebral palsy, Infant, Environmental enrichment, Motor skill

## Abstract

**Background:**

The average age for the diagnosis of cerebral palsy (CP) is 19 months. Recent neuroplasticity literature suggests that intensive, task-specific intervention ought to commence as early as possible and in an enriched environment, during the critical period of neural development. Active motor interventions are effective in some populations, however the effects of active motor interventions on the motor outcomes of infants with CP have not been researched thoroughly, but pilot work is promising. The aim of this study was to determine the short- term effects of “GAME”; a new and novel goal-oriented activity-based, environmental enrichment therapy programme on the motor development of infants at high risk of CP and test study procedures for a randomized controlled trial (RCT).

**Methods:**

Pragmatic 2-group pilot RCT to assess motor outcomes, goal attainment, parent well-being and home environment quality, after 12-weeks of GAME intervention versus standard care. GAME included: creation of movement environments to elicit motor behaviours; parent training in motor learning and task analysis; frequent practice of motor tasks using a programme that was individualised to the child, was varied and focused on self-initiated movement. Data were analyzed using multiple regression.

**Results:**

Thirteen infants were consented, randomised, treated and completed the study. At study conclusion, the GAME group (n = 6) demonstrated an advantage in Total Motor Quotient of 8.05 points on the Peabody Developmental Motor Scale-2 (PDMS-2) compared to the standard care group (n = 7) (p < .001). No significant differences existed between groups on any other measure.

**Conclusions:**

GAME appears to offer a promising and feasible new motor intervention for CP, with favourable short-term motor outcomes. A pressing need exists for an adequately powered RCT with long-term end points, to determine if GAME may advance these children’s motor trajectory.

## Background

Late diagnosis is the norm for children with cerebral palsy (CP) since very few diagnostic biomarkers exists; only half are unwell in the neonatal period [1]; and neuroimaging does not accurately predict severity except in severe cases. This most often leads to a “wait and see” approach, where brain injured babies are monitored but not referred for rehabilitation until marked developmental delay is evident. Formal diagnosis of CP is made on average at 19 months and can be as late as 4 years for those mildly affected, usually after failed motor milestones, or the emergence of clinical signs such as spasticity or involuntary movements. Identifying infants at very high risk of CP early and discriminating them from those with other diagnoses could lead to the provision of more specific, timely and evidence-based CP rehabilitative therapies in the critical period of brain development [1]. Current thinking is that these diagnostic-specific interventions should be applied very early rather than delivering general early intervention (EI), in an effort to optimise outcomes and limit maladaptive plasticity [2,3].

A consequence of the lack of a definitive CP biomarker and late diagnosis is that only a handful of EI clinical trials exist where all participants actually have CP or are at very high risk of CP. Rather, most EI trials comprise of heterogeneous “at risk” populations, including many infants who go on to have normal outcomes, resulting in underpowered trials that do not tell us much about effect of EI in CP [4]. Studies specifically recruiting infants with brain injuries in the newborn period have typically not accurately identified infants who will later go on to be diagnosed with CP and disconcertingly, rarely have the study interventions resulted in motor improvements [5]. Prechtl’s qualitative assessment of general movements (GMs) is the most predictive assessment tool to detect infants, as young as 3 months who have the highest risk of CP, however it is rarely used when recruiting infants to intervention studies [6]. A further confounder in CP intervention studies is the heterogeneity of the condition, creating wide distributions of baseline and change scores making it difficult to detect change and identify best responders and non-responders.

As evidence of the benefits of Environmental Enrichment (referred to as EE from now on) on brain recovery grows [2,5], the focus of CP rehabilitation in older children has shifted towards approaches that emphasise goal-oriented activity-based therapy [7], and frequent task practice with deliberate creation of optimal environments for motor learning. These approaches, based on motor learning principles do not focus on passive interventions such as stretching, or the normalisation of movement like traditional Neurodevelopmental Therapy (NDT), but rather on task practicability and environmental context [8,9]. Improvements in motor behaviour depend upon intentional goal directed practice where the therapist is a “change agent” setting the stage for learning and facilitating the child’s exploration of effective movement solutions [10,11]. Examples of proven effective interventions utilising motor learning principles include constraint induced movement therapy and bimanual therapy. Typically these interventions are offered to children with CP from 2 years of age. Recently, a systematic review and meta-analysis of infants at high risk of CP, showed a small but significant effect of EE interventions on motor outcomes [5], suggesting that diagnostic-specific interventions including EE lead to better outcomes for infants. There remains a significant gap in our understanding of how the motor learning approaches effective in older children with CP can be applied to infants with a very limited motor repertoire. In addition, parent education is known to be an important component of early intervention [12] and since most of the infant’s active practice opportunities are provided within daily routines, parent education and coaching is crucial in order for the necessary practice to take place [13]. We therefore developed a new infant intervention approach: “Goals, Activity and Motor Enrichment” (GAME) that utilized motor learning principles, goal-oriented activity-based therapy, parent education and EE strategies.

The aim of our study was to determine the short-term effects of GAME intervention on the motor development of 3–5 month old infants at very high risk of CP, and to test study procedures in preparation for a Randomised Controlled Trial (RCT). We hypothesized that infants in the GAME intervention group would have higher goal attainment and Peabody Developmental Motor Scale-2 (PDMS-2) scores after 12 weeks of intervention than infants receiving Standard Care (referred to as SC from now on).

## Methods

A pragmatic 2-group pilot RCT was used to explore the feasibility and effects of 12 weeks of GAME (Goals –Activity –Motor –Enrichment) intervention in infants at high risk of CP. GAME intervention is a home-based motor learning approach that aims to advance motor skills of infants and young children via motor task practice, parent education and environmental enrichment. The study also aimed to test the acceptability of randomisation procedures and the intervention to families and referring institutions, and to check outcome measure sensitivity and determine likely effect sizes.

### Study rationale

This study is both an RCT and a feasibility study [14]. We conducted and reported the pilot/feasibility study as an RCT because: 1) we wanted to test whether the randomisation procedure itself was acceptable to referring institutions and parents and therefore it was important to test whether or not it was feasible to recruit participants to an RCT. Since the GMs was new in our locality we were unsure that once the label “high risk of CP” was given to infants whether referral institutions were likely to promote a study where there was equal chance the infant would get a therapy program from a “CP specific” service vs general pediatric therapy programs, which are varied in type and intensity. Moreover, we wanted to see if parents “dropped out” of the study if they were randomised to SC; 2) The intervention was not previously described and we wanted to test the feasibility of both carrying out the intervention and its’ acceptability to parents; 3) The dearth of available outcome measures that are criterion-referenced for infants with disabilities is well established. As Goal Attainment Scaling (GAS) is widely used in toddlers and children with CP we wanted to test whether this was useful with infants who are yet to meet their motor potential; 4) We wanted to test statistical procedures. CP is a heterogeneous condition and the GMs assessment does not predict severity. We expected therefore to recruit infants across the severity levels and for this reason used regression to enable us to account for differing motor ability affecting outcome.

### Participants

Thirteen infants were recruited from 6 Neonatal Intensive Care Units (NICUs) in the Sydney Children’s Hospital Network (SCHN) and from the Cerebral Palsy Alliance, Australia. Infants 3–5 months of age were eligible for enrolment if parental consent was obtained and they had an abnormal GMs assessment score between 11–18 weeks post term age. Since “absent fidgety” GMs are the most predictive of a future diagnosis of CP, we used results from this period [6] rather than the earlier “writhing” period. GMs assessments were scored by at least 2 certified GMs assessors blinded to the infant’s history. No official diagnosis by a medical professional was made at enrolment, rather, parents were counselled about the results of the GMs meaning their baby was at very high risk for CP. Infants were excluded if oxygen dependent, still an inpatient, or lived in a remote location precluding home visits from investigators.

### Procedures

Ethics approval was obtained from the University of Notre Dame Australia, Cerebral Palsy Alliance and the SCHN. After eligibility was determined, informed written consent was obtained and baseline measures taken. Infants were randomised to either the GAME or SC groups using sequentially numbered opaque sealed envelopes. The randomisation sequence was computer generated by an independent officer and group allocation was managed off-site. Intervention was carried out for 12 weeks as per the trial protocol for the 2-groups. Measures were taken at baseline within the child’s home and were repeated at the primary end-point, after 12 weeks of intervention.

### Intervention

GAME: All GAME interventions were provided by the investigators (CM and IN) and carried out within the home environment. GAME has been described elsewhere [15] but always consisted of three components: goal oriented activity-based motor training, parent education, and strategies to enrich the child’s learning environment.

1. Goal-oriented intensive motor training – parent identified goal areas were targeted for practice during the therapy session and after further assessment, a home program (HP), which was a detailed goal focussed activity based home practice plan was devised [16]. The therapist scaffolded all motor tasks, so that the infant could always actively complete at least a part of the task. As performance improved, the challenge was increased by altering the task or environment to a new and appropriate level of difficulty. Manual assistance was provided by the therapist and parent only when necessary for safety or to give the infant the “idea” of the movement. Manual assistance was reduced or withdrawn as soon as the infant demonstrated self-initiated progress with the task; ensuring self-generated motor activity was the focus of all practice. Once a motor skill was learned, variability of practice was introduced to increase the complexity and generalizability of the skill. Early weightbearing and sit to stand from the parents’ lap were part of each HP even if standing was not identified as a specific goal. Rehabilitation research in older children and adults with brain injuries suggest that functional weight bearing exercises can both improve motor control and provide strength training [17]. Given that the expected impairments of CP include weakness and reduced selective motor control, early activation of muscles of the lower limb using both concentric and eccentric exercise could enhance the development of upright mobility. Similarly, practice of reaching and grasping a variety of objects was a standard part of motor training for all infants in order to expose the infants who are expected to be delayed, to a variety of objects to advance grasp and reach behaviours [18].

The written HP was related to parent identified goals, weightbearing and reach and grasp. The HP included photographs, describing parenting strategies, environmental enrichments and child-activities as per published guidelines on effective home programmes [16]. Activities in the HP were organised into those in which the carer played an active role and those where practice could be “set up” for the infant to carry-out independently. The HP was updated once during the 12-week period.

2. Parent Education: Parents were coached to identify their child’s voluntary attempts to move and self-regulate, plus understand the usual trajectory of emergent motor skills and how to stimulate progress. Parents were trained in simple motor task analysis and coached in appropriate strategies to enhance their child’s development both at a specific goal level and in general early learning and development principles. Parents were taught to optimise the best use of their infants’ awake time and the naturally occurring opportunities for learning. Learning optimisation included both parent-directed and structured practice of desired motor tasks, where the parent role was integral to the child’s learning (e.g. creating repetitions) and constructing opportunities for independent play (e.g. playing alone with motor enriching toys set up for the child). Parents were encouraged to both observe the therapist eliciting a motor behaviour from the baby and to attempt it themselves. Specific feedback was given to parents to enable them to tease out why some attempts were successful for the baby and others weren’t. As new motor skills emerged parents were coached in strategies to increase the challenge of the task; for example remove support or introduce a more complex toy. The importance of allowing trial and error during practice was discussed and parents were encouraged to devise their own activities to enhance goal attainment.

3. Environmental Enrichment – Parents were encouraged and assisted to set up motor enriched play environments to promote child self-generated movements, exploration and task success. This included instruction in careful toy selection “matched” to the desired motor task, plus physical set up of areas for practicing and repeating activities related to the identified goal areas, weightbearing, and reaching and grasping tasks. Conventional baby equipment (e.g. highchairs, toys) already purchased by the family was used wherever possible. The whole environment for motor learning was taken into account and therefore intervention also included: (a) evidence-based early learning stimulation and role modelling to enhance cognitive and language development (e.g. reading books to children, limiting passive television watching); (b) optimising sleep hygiene, for example assisting with implementing sleep routines; and (c) feeding interventions (e.g. anti-reflux medications) to ensure adequate caloric nutrition and pain-free backdrops for learning. The importance of variable daily experiences for infants was deliberately addressed and support given when parents articulated difficulty leaving the house. Siblings and extended family members were also actively encouraged to take part in the HP and therapy sessions to promote: family knowledge; family acceptance; family wellbeing; repetition of learning opportunities; and provide a natural source of varied social interaction for the infant.

Intervention was customised for the child’s motor ability, the family enrichment style, and parent goals. Therapist visits were weekly initially and then frequency was negotiated with each family around their preferences, availability and parental skill level to carry out GAME with fidelity. Visits typically lasted for 60 to 90 minutes.

Standard Care: Therapy intervention for infants at high risk of CP is available in New South Wales (NSW) free of charge, upon medical referral but varies enormously with no gold-standard guidelines in existence. Prior to study commencement, a survey was conducted amongst the study recruiting sites, revealing that the intensity of SC therapy was an average of 14-hours in the first year of life, spread typically over fortnightly or monthly appointments. Not all NICU recruitment sites offered ongoing intervention and referred infants to community-based organisations. The content of SC typically involved physical guidance to facilitate normal movement patterns and parental advice on positioning and handling. As no employer guidelines exist the choice of therapy approach is decided by the treating therapist and might have included NDT, motor learning, the developmental skills approach or a combination of approaches. For study purposes the SC offered to the control group was outside the investigators control both in terms of type of therapy and intensity of therapy, but was however representative of SC. Infants randomised to SC were referred to the provider by the centre referring the infants to the study. Infants received SC from either a hospital (n = 2), a community-based health centre (n = 3), or a Not-For-Profit Organisation (n = 2).

### Outcome measurement

The primary outcome measure was Goal Attainment Scaling (GAS), an individualised criterion-referenced measure of goal performance. Goals are set, with five possible outcomes specified for each goal. Composite T-scores are calculated for multiple goals and change over time is quantified using change scores and using conventional procedures recommended in literature [19]. We treated GAS scores as a continuous variable rather than ordinal although both approaches are used in the field and disagreement exists [19]. GAS is useful in CP rehabilitation for detecting incremental change in functional abilities that might not be detected on norm-referenced tools such as the Bayley Scales of Infant and Toddler Development [20]. GAS is widely used and recommended in childhood CP research because it is valid, reliable and responsive [19]. The use of GAS to measure outcomes in infants with CP has been validated [21] but never used in RCTs of infants under 12 months of age with limited motor repertoires and thus sensitivity is untested for this younger population. We therefore wanted to test the usefulness and applicability of GAS in very young infants across a broad spectrum of motor ability. We used GAS because we wanted to capture incremental change in performance. At the initial appointment after consent had been obtained, parent identified functional developmental goals for their child from interview. These were formulated into individual goal scales prior to the commencement of therapy with the baseline level set by the investigators on the basis of an initial assessment of ability of the identified goal and confirmed by parent interview. GAS banks have been recommended in literature as a way of improving rigour. We used GAS banks wherever possible but individualised the goals as per the tool conventions when banks did not exist. For example, if the same baseline ability was evident for different participants for a specific goal the same GAS levels from a bank were used. As per test developer conventions parents were encouraged to identify 3 to a maximum of 5 goals for the 12-week period. Assessors were blinded to group allocation and scored the infant’s 12-week GAS performance from video.

#### Canadian Occupational Performance Measure (COPM)

The COPM is an individualised, criterion referenced tool measuring perceived change in infant performance and parental satisfaction with performance over time on family priorities. The COPM is widely used in CP research and is valid, reliable and responsive [8,22]. During a semi-structured interview parents identified a number of areas that they would like to focus on with their baby during the study period. The standard 10-point scale was used to rate the infant’s performance and their own satisfaction with the infant’s performance on the identified focus areas. This was repeated after 12-weeks by a blinded assessor. An improvement of two or more points is regarded as clinically significant [22].

#### Peabody Developmental Motor Scales - Second edition (PDMS-2)

The PDMS-2 [23] is standardised norm-referenced tool, which is valid, reliable, and widely accepted. A total of 5 sub-scales are assessed including reflexes, locomotion, stationary, grasp and visual motor integration. A total motor quotient (TMQ) is calculated with a mean of 100 and SD of 15. Responsivity has been established for infants for the original version [24] and for toddlers with CP for the PDMS-2 [25]. The PDMS-2 was selected preferentially over the gold standard Gross Motor Function Measure (GMFM) because it evaluates fine motor skills that are targeted in many early intervention programmes.

#### Home Observation Measurement of the Environment (HOME) - infant-toddler version

The HOME [26-28] is a reliable, valid standardised measure of the quality and quantity of parent and home environmental stimulation and support available, scored from parent interview and direct observations. Sub-scales include parent responsivity, the availability of learning materials and variety of stimulation. The infant – toddler version is suitable for ages 0–3 [26]. Higher total HOME scores indicate a more enriched environment with 45 being the highest possible score.

#### Depression, Anxiety and Stress Scale (DASS-21)

The DASS-21 [29] is a mental health self-report measure of the emotional states of depression, anxiety and stress. The DASS-21 is psychometrically sound and is useful tool in the postnatal period for assessing psychological risks [29]. The primary caregiving parent completed the DASS 21 at baseline and study completion.

#### Logbooks

All families were asked to complete a logbook of the number and length of therapy sessions received over the 12-week study period. Families also documented the amount of time they spent carrying out therapist recommendations in the home environment. Parents who chose to access additional therapist-provided intervention documented the number of extra sessions.

### Statistical analysis

Parent and infant characteristics and baseline measure mean scores were compared using independent t-tests, to ensure baseline equivalence of groups. Linear regression was used (where baseline scores were entered as covariates) to test the effect of providing GAME intervention compared to SC, on the infant’s goal attainment and motor performance, the home environment and the parent’s mental health. We chose to use linear regression over traditional t-tests as CP is known to be a heterogeneous condition and we expected to recruit infants across the severity spectrum leading to a wide variety of baseline scores and large standard deviations in both groups. Linear regression allowed us to treat baseline scores as a covariate. Severity could not reliably be imputed as a covariate in this short duration, small sample study, although this would be highly desirable, because 42% of infants change severity levels on the gold standard scale under 2-years of age [30]. Post-hoc analysis of the effect of total therapy dose (therapist delivered intervention plus parent delivered home program practice) in hours on the outcome was also conducted because there was insufficient power to use intensity of therapy as a covariate in the regression. Analyses were conducted on the basis of intention to treat. Missing values were imputed as last observation carried forward. Results were presented as between group differences with 95% confidence intervals.

Effect size was computed using Cohen’s d. Commonly used criteria specify that a value below 0.2 is regarded as no effect, a value of 0.2–0.5 is a small effect, a value of 0.5–0.8 is a medium-sized effect and a value above 0.8 is a large effect [31].

## Results

**Figure 1:**
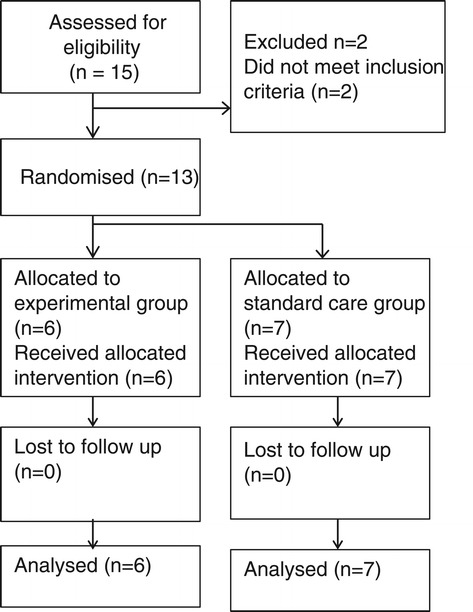
**Flow of participants.**

Thirteen infants from twelve families, mean age 17.6 weeks (SD =3.9), corrected for prematurity, and at very high risk of CP were recruited between September 2011 and September 2012 (Table 1). Six infants were randomised to the GAME and seven to SC. Twins were randomised into the same group, as it would be impossible for parents to operationalize two different treatment approaches without intervention contamination. The flow of participants through the study is summarised in Figure 1. Adherence to study protocols was excellent with no dropouts. Participant characteristics are summarised in Table 1. Groups were equivalent at baseline on infant and parent characteristics. All child outcome data was normally distributed at both baseline and follow-up, therefore meeting the assumption for parametric statistics. The only exception to this was the HOME follow-up data, which was skewed right (kurtosis of 3.24) indicating ceiling effects on the measure.

**Table 1:** **Baseline characteristics of participants**

**Characteristic**	**GAME (n = 6)**	**Standard care (n = 7)**	**p value**
Gestational age, mean (SD), weeks	35.50 (5.21)	33.57 (7.76)	0.61
Age at baseline, mean (SD), weeks (corrected for prematurity)	17.83 (4.17)	17.43 (3.95)	0.86
Sex: M/F	5/1	6/1	-
Birthweight, (kg)	2.85 (1.19)	2.40 (1.40)	0.54
Parent age, years			
Mother	33.00 (3.34)	33.43 (5.0)	0.86
Father	39.17 (5.12)	38.43 (2.64)	0.76
GAS T-score, mean (SD)	21.50 (1.22)	22.43 (0.96)	0.47
COPM Performance score, mean (SD)	3.03 (1.01)	3.19 (0.58)	0.42
COPM Satisfaction score, mean (SD)	4.26 (0.89)	4.81 (1.31)	0.36
PDMS-2			
Total Motor Quotient	80.17 (8.98)	81.29 (9.20)	0.83
Total Motor Standard Score, mean (SD)	35.67 (6.56)	36.43 (6.88)	0.87
HOME – IT score, mean (SD)	33.83 (3.66)	29.00 (8.08)	0.06
DASS 21 score, mean (SD)	19.67 (8.71)	24.57 (23.96)	0.16
Risk for CP*			
• Premature			
<28 weeks	n = 1/6	n = 3/7	-
>28 - < 37 weeks	n = 1/6	n = 0/7	-
• HIE	n = 2/6	n = 3/7	-
• Multiple Birth	n = 2/6	n = 0/7	-
• Hydrocephaly	n = 0/6	n = 1/7	
Absent Fidgety General Movements Score (12–16 weeks PTA)	n = 6/6	n = 7/7	-
Diagnosis of CP between 5-12months	n = 4/6	n = 6/7	-

*Primary risk factor - some participants had >1 risk factor. GAS = Goal Attainment Scaling; COPM = Canadian Occupational Performance Measure; PDMS-2 = Peabody Developmental Motor Scales – second edition; HOME = Home Observation Measurement of the Environment; DASS 21 = Depression, Anxiety, Stress Scales short (21 item) version; HIE = Hypoxic Ischaemic Encephalopathy; PTA = post term age.

### Primary outcome at the primary end-point – GAS at 12 weeks

Primary and secondary outcomes are presented in Table 2. After 12-weeks of intervention, both groups improved. The mean change score for GAME intervention was 38.67 (SD = 7.63) and 28.28 (SD = 18.33) for the SC group but with no statistically significant between-group differences and wide variation about the SC mean. Infants in both groups achieved the expected motor outcomes for parent-identified therapist-set goal scales (Table 2), improving 2 SDs from baseline on GAS T-Scores (GAS mean T-score = 50, SD = 10, with a T-Score 40–60 indicating achievement as expected). Parents usually identified 4–5 motor goals for their infants including rolling (77%), sitting (54%), reaching in prone (54%) and grasping toys (54%). One parent identified a non-motor goal (improved sleeping).

**Table 2:** **Primary and secondary outcome measures with estimates of effect (between group differences and 95% confidence intervals)**

**Outcome**		**Group**		**Estimate of effect (95% CI)**	**p-value**
**Time Point**	**Measure**	**GAME (n = 6)**	**SC (n = 7)**		
		**Mean (SD)**	**Mean (SD)**		
**Infant goal achievement on motor tasks:**					
Baseline	GAS T-Score	21.50 (1.22)	22.43 (0.98)	-	-
12-weeks	GAS T-Score	60.17 (6.62)	50.71 (18.33)	7.37 (−12.71, 27.45)	0.43
**Parent perception of infant motor performance-**					
Baseline	COPM Performance	3.03 (1.01)	3.19 (0.58)	-	-
12-weeks	COPM Performance	7.24 (1.11)	6.58 (2.10)	0.72 (−1.49, 2.92)	0.49
Baseline	COPM Satisfaction	4.26 (0.89)	4.81 (1.31)	-	-
12-weeks	COPM Satisfaction	7.42 (1.05)	7.49 (2.56)	0.13 (−2.54, 2.79)	0. 92
**Parent enrichment style**					
Baseline	HOME Score	33.83 (3.66)	29.00 (8.08)	-	-
12-weeks	HOME Score	39.83 (2.14)	36.43 (6.90)	−0.13 (−3.48, 3.22)	0.93
**Infant motor development**					
Baseline	PDMS-2 TMQ	80.17 (8.98)	81.29 (9.20)		
12-weeks	PDMS-2 TMQ	84.67 (10.21)	77.71 (8.85)	8.05 (3.88-12.23)	<0.00*
Baseline	PDMS-2 Total motor SS	35.67 (6.56)	36.43 (6.88)	-	-
12-weeks	PDMS-2 Total motor SS	38.83 (7.44)	33.86 (6.44)	5.72 (2.88, 8.56)	.001*
**Parent well being**					
Baseline	DASS 21 Total	19.67 (8.71)	24.57 (23.96)	-	-
12-weeks	DASS 21 Total	13.67(11.83)	26.00 (28.75)	−7.49 (−24.86, 9.89)	0.36

GAS = Goal Attainment Scaling; COPM = Canadian Occupational Performance Measure; PDMS-2 TMQ = Peabody Developmental Motor Scales – second edition Total Motor Quotient; PDMS-2 total motor SS = Peabody Developmental Motor Scales – second edition total motor standard score; HOME = Home Observation Measurement of the Environment; DASS 21 = Depression, Anxiety, Stress Scales short (21 item) version.

*Indicates statistically significant.

### Secondary outcome measures

PDMS-2: After 12 weeks of intervention, the infant’s motor abilities were assessed using the PDMS-2. Statistically significant between group differences were found in the Total Motor Quotient (TMQ) PDMS-2 scores, conferring an 8.05 point advantage to the GAME intervention group (95% CI 3.88-12.27; p < 0.001). This represents just over 0.5 of a SD on the PDMS-2, which is probably clinically significant based on Wang’s calculation for toddlers [25], but since no data on clinically meaningful change exists in infancy we cannot be certain. The total composite motor scores are also provided in Table 2 but the primary analysis was conducted on the TMQ because it is regarded as the most psychometrically robust estimation of motor ability.

We calculated sensitivity to change coefficients using Cohen’s effect size, to assist with interpretation of the results. The Cohen’s effect size for the GAME group was 0.5, which is considered a small to moderate effect size, while the SC group was −0.4, which Cohen defines as trivial since the change is <0.2.

COPM: COPM performance and satisfaction scores improved in both groups with no between-group statistical differences.

HOME: Scores on the HOME improved in both groups however there were no statistically significant between group differences.

DASS 21: DASS 21 scores were calculated for 12 mothers and 1 father, with no between-group statistical differences found. Mean DASS 21 scores dropped in the GAME group by 13.67 points (SD = 11.83) but were stable in the SC group with an endpoint mean of 26.00 (SD = 28.75). The large SD in the SC group is explained by the scores of one parent who had a pre-existing severe mental health condition.

Logbook: Adherence to the GAME study protocol was high for all families. All GAME parents completed the logbook indicating HP and therapy time. All families in the SC group recorded therapy visits however 2/7 did not record HP time. These were the only missing values in the analysis and were coded as missing. Seven of the 13 infants were formally diagnosed with CP during the study period. Another 3 were formally diagnosed by 12 months and the developmental outcome of another 3 is unknown (2 in GAME group and 1 in SC). No information was collected about the type or severity levels of those diagnosed in this small pilot study.

Post-hoc analysis of the dose of therapy found a significant difference between groups in both the number of hours of therapy and the numbers of hours HP time. Infants in the GAME group received an average of 9.93 (range 7.5-15 hours) hours of therapy, which was almost three times higher than the 3.49 hours (range 1–6 hours) received by the SC group (p < 0.00). Parents in the GAME group also spent more time carrying out the HP. The mean total dose of therapy (therapy plus HP) was 140.58 hours (SD 23.3) for GAME, and 54.17 hours (SD 32.62) for SC.

## Discussion

We hypothesised that GAME infants would have higher GAS scores than SC infants. Mean GAS score for the GAME group was a full GAS T-Score SD higher than that of the SC group. Statistical significance was not reached but this was not expected in this feasibility RCT which was underpowered to detect change, leading to a probable type II error. Interestingly goal achievement was higher and more homogenous in the GAME group whereas great variation was evident in SC scores, perhaps indicating GAME was more goal-focused - an issue that could be further examined in future studies. We also noted that therapists found it difficult to predict the rate of infant’s motor development at baseline given the limited motor repertoire at enrolment age and the lack of a robust severity measure for infants. Prior to intervention when goals were set, parents had difficulty predicting their baby’s rate of development and their knowledge of what was “normal” varied. For example some parents did not know when a child would normally sit or walk. Parents were taught information in the parent education component of GAME but at baseline knowledge of milestone attainment affected levels of parental concern and GAS prediction accuracy. Although GAS has been shown to be an effective measure of motor change for infants [20,21] it might be more useful for documenting incremental change rather than standard milestone acquisition within clinical trials. We concluded that whilst GAS is sensitive in older children, the parent and therapist inaccuracy of predicting infant motor outcomes substantially affected sensitivity and therefore we would not recommend using GAS as a primary outcome in our own future GAME studies with infants.

Although this study was a small pilot randomised trial the secondary findings suggest that 12 weeks of GAME intervention might have a beneficial effect on the developmental motor outcomes of infants at high risk of CP. There have been no publications on the PDMS-2 about how much change is required in terms of motor quotients or raw score points to be regarded as clinically meaningful in this very young population. However, Wang et al. suggested a change of more than 9 raw score points on the PDMS −2 may be clinically significant [25] amongst toddlers. Our data exceeded the 9 points for all participants but was even greater for the GAME group, however this is a period of rapid motor development so greater change is expected, limiting interpretation of our results. While infants in both groups demonstrated improvements in terms of goal attainment, TMQ scores at 12 weeks on the PDMS-2 were significantly better in the GAME group. This difference could be the result of intensity alone or possibly a result of both the type and intensity of the intervention, as GAME parents engaged in more practice at home than did SC parents. Although the PDMS-2 motor gain is pleasing in this study, children with a permanent physical disability like CP usually fall further behind peers as developmental motor expectations increase. We would therefore expect that for a study of longer duration, the TMQ would drop in children with CP even if raw scores continued to increase. The small-moderate effect size we found in this pilot therefore needs to be confirmed in a larger sample of children over a longer period of time.

The lack of significant between-group differences on the subjective COPM was surprising given that the GAME groups scored better on the PDMS-2. This result might indicate that parents of infants at high risk of CP are pleased with any noticeable improvement or with natural developmental gains, and do not expect age appropriate performance or do not know what motor skills are considered “normal” at various time points. Most parents expressed a general goal for their child to “develop normally” although they were not sure what developmental milestones they should precisely expect. Even though the COPM and GAS scores did not demonstrate significant differences, we found the goal-oriented approach framed by these tools assisted parents to be more specific in identifying concerns, thus enabling focussed HP practice.

Environmental enrichment as measured by HOME scores demonstrated gains in both groups but there were no significant between-group differences. Notably ceiling effects existed, with 9/13 participants having higher than average baseline scores. Previous HOME studies have confirmed this ceiling effect [32]. It should be noted that the baseline HOME scores of the SC displayed a higher degree of variance than the GAME group due to 3 families with scores below the published mean of 31 (26) and only 1 in the GAME group. However after 12 weeks only one family in the SC group still scored below the mean. Future GAME studies should endeavour to explore the use of other measures of EE that might be more sensitive to change.

DASS 21 scores between groups were comparable at baseline and after intervention. At baseline, 23% of parents (all mothers) had abnormal depression scores but after intervention this had dropped to 15%. Miller at al [29] reported a DASS 21 depression rate of 19% in primiparous mothers, so our result was not surprising as mothers in the study experienced additional stressors in the newborn period. At baseline 31% of parents (all mothers) had symptoms of anxiety and this had reduced to 15% after 12 weeks of intervention. Our sample’s baseline anxiety rate was higher than previously reported rate of 13% in new mothers. Premature birth and exposure to intense medical environments such as Neonatal Intensive Care Units are known risk factors for adverse psychological symptoms in mothers [33]. Adaptation to the diagnosis of CP is another known stress point and families participating our study were at risk of poor emotional health because of these factors. Evaluating parent wellbeing in studies of infants at high risk of CP is important as parental depression and anxiety can affect parent-infant attachment [33], negatively influence child cognition [34] and might impact the mother’s ability to carry-out HPs.

### Feasibility of the trial

We found GAME was both feasible to carry out and acceptable to parents and referrers, with no dropouts, minimal missing data, and only n = 1 parent declining to enrol. Ten of 12 families completed the logbook of HP and two forgot, but were able to estimate data. Although some described the logbook as tedious, it provided invaluable information about dose of practice.

GAME intervention fidelity was maintained as the same therapists provided intervention for each infant in the GAME group. Intensity of SC intervention was variable and little information was available about the type of SC intervention. Future studies should attempt to describe the content of SC more specifically.

The pilot study enabled us to confirm outcome measures for a planned larger RCT and calculate the sample size required with PDMS-2 as the primary outcome measure.

### Limitations

There were several limitations to this pilot study. First, the small sample size gives rise to the possibility that the absence of GAS, COPM and HOME differences could be type II errors arising from low statistical power. Second, the study period was relatively short and infants were only 6–8 months old at the primary endpoint. It is therefore not clear whether the advantage observed in the GAME group would have been maintained long-term, particularly since at one-year of age the more demanding motor tasks of upright ambulation is the developmental norm. Third, as previously discussed, it is possible that the higher PDMS-2 GAME scores might have been solely attributable to the dose of therapy rather than GAME intervention. Dose of therapy will be entered as a covariate in the planned larger trial, however GAME intervention itself may in fact lead to greater parental participation in home practice as parent education is regarded as a key component of the intervention. Fourth, since SC is variable, areas of overlap in approach could well have existed creating contamination between the groups. Fifth, the lack of evaluator blinding across some measures may have unintentionally led to observer bias.

A larger blinded, RCT of infants from 3 months to one year is required to investigate whether the benefits of GAME confers a similar result to this pilot long-term. We did not find GAS the most appropriate primary measure to use in an RCT with young infants, and recommend a suite of measures including both a norm referenced tool complemented by criterion referenced measures capable of detecting incremental motor change, such as the COPM and GMFM. Future studies with larger sample sizes should also treat severity of motor impairment and dose as covariates in the analyses.

## Conclusions

This pragmatic pilot study compared 12 weeks of goal-oriented, activity-based, motor training centred on parent-elicited goals (“GAME”) to SC in infants at high risk of CP. While infants in both groups attained their goals, GAME infants had higher scores on a standardised assessment of motor ability, providing preliminary promising evidence of efficacy of GAME. Parent reported improvement in COPM performance and satisfaction and home enrichment scores improved in both groups. Mothers tended to report higher depression and anxiety scores than mothers without infants with a disability, indicating parental well-being is important to monitor. The recruitment processes and intervention was clinically feasible to do and acceptable to all families.
